# The Role of LC-MS in Profiling Bioactive Compounds from Plant Waste for Cosmetic Applications: A General Overview

**DOI:** 10.3390/plants14152284

**Published:** 2025-07-24

**Authors:** Gilda D’Urso, Alessandra Capuano, Francesca Fantasma, Maria Giovanna Chini, Vincenzo De Felice, Gabriella Saviano, Gianluigi Lauro, Agostino Casapullo, Giuseppe Bifulco, Maria Iorizzi

**Affiliations:** 1 Department of Pharmacy, University of Salerno, Via Giovanni Paolo II 132, 84084 Fisciano, Salerno, Italy; gidurso@unisa.it (G.D.); acapuano@unisa.it (A.C.); glauro@unisa.it (G.L.); bifulco@unisa.it (G.B.); 2 Department of Biosciences and Territory, University of Molise, Contrada Fonte Lappone, 86090 Pesche, Isernia, Italy; fantasma@unimol.it (F.F.); defelice@unimol.it (V.D.F.); saviano@unimol.it (G.S.)

**Keywords:** plant waste, LC-MS analysis, metabolomics, cosmetic applications, bioactive compounds

## Abstract

The agro-industrial sector produces large amounts of by-products that have a high environmental impact, so it has become essential to recover food waste at all levels. This is because it often contains bioactive molecules that can be a valuable source of new products such as animal feed, biopolymers, or products for human use, (e.g., cosmetics and nutraceuticals) due to its antioxidant, antimicrobial, and anti-inflammatory properties. Advanced analytical methodologies such as liquid chromatography coupled to mass spectrometry (LC-MS) are crucial for the characterisation of bioactive chemicals in these waste materials. LC-MS enables both targeted and untargeted metabolomic approaches, facilitating the identification and quantification of a wide range of secondary metabolites, including polyphenols, flavonoids, alkaloids, and terpenoids. The choice of extraction methodology is essential for the precise identification and quantification of these metabolites. This study provides an overview of LC-MS as an effective tool for analysing complex extracts derived from plant waste, discussing both methodological aspects and typical bioactive metabolites identified, and offering examples of their potential applications in cosmeceutics.

## 1. Introduction

In recent years, the problem of industrial and plant waste, with its significant environmental and economic implications, has gained increasing attention [1,2,3]. The most effective method of managing bio-waste is its transformation into new resources, such as animal feed and organic fertilizers, or its reintegration into several industrial sectors and conversion into high-value products [4]. Many companies are increasing their interest in this area and, beyond the most common agricultural and food sectors, even the cosmetic industry is adopting this approach, helping the reduction of CO_2_ emissions and promoting sustainable consumption.

Cosmetics production could significantly benefit from the potential of these by-products [5], since food waste materials, such as seeds, peels, leaves, and other vegetal-derived parts, are rich in bioactive compounds, despite usually being discarded [6,7]. They often contain secondary metabolites that possess antioxidant, anti-inflammatory, antimicrobial, and even anti-aging properties. Such characteristics are particularly relevant for skincare, personal care formulations, or other beneficial products that are highly relevant in the field of cosmetics, where natural ingredients (e.g., polyphenols, flavonoids, vitamins) are increasingly preferred over synthetic alternatives [8].

Some interesting applications of food waste in cosmetics have already been reported, highlighting the great potential of their repurposing. For example, the analysis of by-products obtained from the pruning of orange, apricot, and olive trees led to the identification of antioxidant compounds that were then used to formulate a stable, consistent, and pathogen-free cosmetic cream [9]; moreover, pequi oil, extracted from the Brazilian fruit *Caryocar brasiliense*, offers moisturising, antioxidant, and anti-aging properties [10]; in addition, clementine peel and olive leaf extracts, obtained via eco-friendly supercritical extraction, can be safely and effectively incorporated into stable, antioxidant-rich cosmetic creams, promoting sustainable skincare solutions [11].

Thus, characterising bioactive compounds in such waste matrices is crucial for determining the best way to exploit them.

In this context, advanced analytical techniques such as liquid chromatography coupled to mass spectrometry (LC-MS) play a crucial role. By combining the separation power of liquid chromatography with the high sensitivity, specificity, and broad detection capabilities of the MS technique, it is possible to achieve sensitive and accurate profiling of complex metabolite mixtures [12]. Through both targeted and untargeted metabolomic approaches, LC-MS enables the identification and quantification of novel compounds and known bioactive components, providing insight into their chemical properties and biological potential.

**Figure 1:**
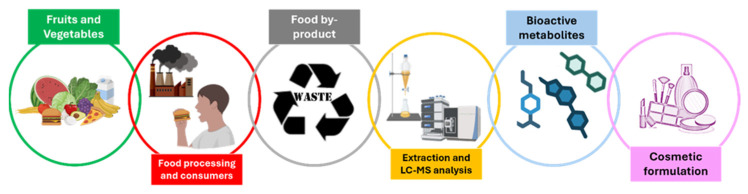
Valorisation of agro-industrial by-products through their integration into advanced cosmetic formulations, contributing to waste minimisation and sustainable resource utilisation.

The correct choice of the extraction procedure is, of course, critical for the accurate identification and quantification of these compounds through LC-MS (Figure 1). The development of eco-friendly approaches is closely linked to effective and selective extraction techniques following green chemistry principles. Conventional extraction processes often involve the use of hazardous organic solvents and energy-intensive methods, whereas green extraction processes provide more sustainable alternatives [13,14,15]. These green approaches include ultrasound-assisted extraction (UAE), microwave-assisted extraction (MAE), pressurised liquid extraction (PLE), and the use of bio-based solvents or supercritical fluids [16,17,18].

This review examines the potential of LC-MS analysis for characterising bioactive compounds in green extracts derived from fruit and vegetable waste. Particular attention is given to both the analytical methodologies involved and the classes of metabolites typically identified through targeted and untargeted approaches from plant waste. The study also highlights the relevance of these compounds for the development of cosmeceutical formulations, emphasising their functional properties, including antioxidant, antimicrobial, and anti-inflammatory activities.

## 2. Application of Liquid Chromatography for Metabolite Profiling in Plant Waste

Plant waste matrices represent a great source of bioactive compounds with potential applications in the cosmetic industry. However, the complex nature of waste matrices represents a substantial problem for metabolite analysis. The heterogeneity of these materials results in complex extraction mixtures that contain a plethora of structurally diverse compounds, spanning a broad concentration range, which makes them challenging to separate, identify, and quantify.

Liquid chromatography is a method that enables the separation of various components in a mixture based on their different structural characteristics. The classification is based on their mode of separation, using normal phase (NP-LC), reversed phase (RP-LC), size exclusion (SEC), and ion exchange (IC-LC) stationary phases. The direct interaction between the stationary phase and metabolites occurs through affinity, size, or electrostatic interactions, and different behaviours of the molecules towards the stationary phases will determine their separation [19].

Reverse phase liquid chromatography (RPLC) is commonly employed in phytochemical metabolome [20,21] profiling, based on the use of a non-polar stationary phase (usually C18 or pentafluorophenyl core shells) and several polar solvents as the mobile phase (water and organic solvents like methanol or acetonitrile). The different constituents of a chemical mixture will interact with the stationary phase and then be eluted by the mobile phase according to their polarity. The mobile phase crosses the column through a gradient, starting from solvent mixtures rich in polar solvents to mixtures rich in non-polar solvents. Thus, compounds are sequentially separated, with the most polar molecules eluting first and arriving at the mass spectrometer prior to the less polar ones. To date, this method has significantly evolved through the years with the introduction of techniques like high-performance liquid chromatography (HPLC), ultra-high-performance liquid chromatography (UHPLC) and ultra performance liquid chromatography (UPLC), with the reduction of the particles size in the stationary phase (3–5 μm, 2 μm and <2 μm respectively) providing higher resolution, shorter processing times, and increased sensitivity [22,23].

Additionally, hydrophilic interaction liquid chromatography (HILIC) has emerged as a valuable technique for the analysis of natural matrix extracts [24], allowing the analysis of those compounds that elute too fast during an RP-LC run. It utilises polar stationary phases and organic solvents as the mobile phase, making it suitable for the separation of polar and semi-polar compounds, such as polyphenols, alkaloids, flavonoids, phenolic acids, catechins, and anthocyanins [25,26,27]. Over the years, HILIC has been associated with environmental concerns, leading to the utilisation of solvents (water/ethanol/CO_2_) that enable the separation of sustainable compounds [28].

HILIC is the optimal choice for the retention and separation of polar compounds, but it presents some limitations in the analysis of non-polar compounds. Although RPLC offers excellent peak resolution and wide applicability, it can be challenging to use with highly hydrophilic and polar analytes. A sophisticated strategy for overcoming the limitations of both techniques is to combine them in a two-dimensional setup (HILIC × RPLC), since the two dimensions can separate different classes of compounds. Thus, they offer high orthogonality, providing better separation and better compound identification [19,29].

## 3. Mass Spectrometry for Plant Waste Metabolome Analysis

A variety of analytical methodologies are used to analyse the metabolome of plant samples, such as nuclear magnetic resonance (NMR), ultraviolet-visible spectroscopy (UV-Vis), capillary electrophoresis (CE). Liquid chromatography coupled with mass spectrometry (LC-MS) and, in particular, liquid chromatography coupled with tandem mass Spectrometry (LC-MS/MS) has great advantages in the detection of trace compounds and the elucidation of their molecular structures [30,31,32]. The high sensitivity of LC-MS enables the detection of compounds present at very low concentrations, providing the opportunity to work with high resolution and selectivity, which allows for the precise identification and structural characterization of a wide range of metabolites, including those that are isomeric or structurally similar [33].

After chromatographic separation, the eluted metabolites are transferred to the mass spectrometer, where they undergo ionisation and are then analysed based on their mass-to-charge ratios. A critical factor that influences the success of metabolomic analysis, beyond the LC separation efficiency, is the choice of ionisation source, as it directly affects ionization efficiency and, consequently, detectability. Therefore, selecting the appropriate ionization method is essential to ensure comprehensive metabolite coverage and reliable data interpretation.

Common ionisation sources that can be coupled with an LC system include electrospray ionization (ESI), atmospheric pressure chemical ionization (APCI), and atmospheric pressure photoionization (APPI).

ESI is a soft ionisation source suitable for high molecular weight compounds, as it can produce multi-charged ions. The ionisation step occurs in nanodroplets and can take place in either positive or negative mode, generating protonated molecules [M+H]^+^ or deprotonated molecules [M−H]^−^. The choice of ionisation mode depends on the solution phase and the pKa of the compounds. ESI is applicable for soluble and polar molecules, making it a highly selective ionisation source. This selectivity enables the detection of polar compounds present in low concentrations within complex organic mixtures. For example, a QTOF instrument coupled with an ESI source operating in negative ionisation mode was used to analyse bioactive compounds extracted from avocado peels, to explore their potential applications in the food and nutraceutical industries. The analysis led to the identification of phenolic acids, flavonoids, phenylethanoids, and lignans [34].

Nevertheless, in the APCI source, analytes are volatilised at high temperatures and ionised through proton transfer or charge exchange, depending on the solvent type and the physicochemical properties of the compounds. It is a robust and reliable technique commonly used for the analysis of small polar and nonpolar compounds, as well as thermally stable compounds with low ESI response. Another versatile mass spectrometry ionisation technique, ideal for analysing nonpolar and low-polarity chemicals that are not suitable for ESI, is APPI. Plasma lamps with inert gases, such as krypton or xenon, generate vacuum ultraviolet (VUV) photons for gas-phase operation. These photons generate radical cations or protonated species. The low chemical noise, large dynamic range, and minimised matrix effects of APPI are also notable. In environmental and petrochemical investigations, it outperforms APCI in detecting specific constituent classes in complicated samples like crude oil and biomass [34].

### 3.1. Untargeted and Targeted Approaches in Metabolomics

**Figure 2:**
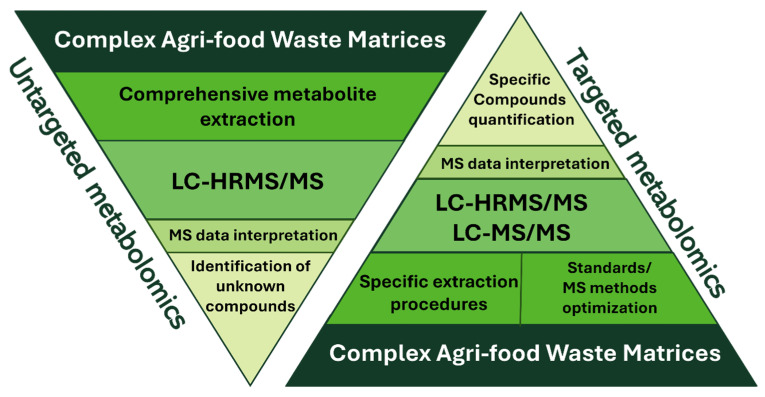
Schematic representation of targeted and untargeted metabolomics workflows.

After liquid chromatography separation and ionisation in either positive or negative mode, metabolites are sent to the mass analyser, and, if necessary, isolated based on their mass-to-charge ratio (*m*/*z*). In the context of metabolome analysis, there are two main approaches to mass spectrometric analysis: untargeted and targeted metabolomics (Figure 2).

Untargeted metabolomics aims to detect as many metabolites as possible within a sample, including those that are unknown, while targeted metabolomics focuses on the selective and sensitive analysis of specific classes of metabolites. Both approaches have distinct advantages and limitations, and they are often used in a complementary manner to achieve comprehensive detection and accurate quantification of a wide range of metabolites [35]. Both strategies are based on different applications of tandem mass spectrometry (MS/MS), specifically the selection and fragmentation of a precursor ion to produce a fragmentation that provides structural information. In addition to traditional MS/MS, enhanced multistage mass spectrometry (MS^n^) enables the sequential fragmentation of product ions, revealing the structures of complex metabolites. It is particularly helpful in untargeted plant waste metabolomics, as compound identification often involves unknown or structurally related compounds. MS^n^ requires ion traps to isolate and fragment ions in several stages (MS^3^, MS^4^, etc.) [36].

#### 3.1.1. Untargeted Analysis

High resolution is a fundamental requirement for a reliable and comprehensive analysis of a species’ metabolome. It allows the discrimination of compounds whose mass-to-charge (*m*/*z*) ratios differ by only a few decimal places, which is crucial in untargeted metabolomics, where thousands of metabolites with similar structures coexist, particularly in complex matrices such as plant tissues or agri-food waste. This is why two different types of mass analyses are commonly employed in metabolome analysis: the Orbitrap and the time-of-flight (TOF). TOF instruments allowed Rodríguez-Blázquez et al. to characterise the total phenolic and flavonoid content profile of plum (*Prunus domestica* L.) seed residue extracts [37].

These analysers are significantly more successful thanks to their integration into hybrid instrument geometries. The hybrid Q-Orbitrap (Q-Exactive and Orbitrap Exploris) and Q-TOF (Xevo G2-XS and G3-XS QTOF, Q-TOF 6545/6546) deliver high-resolution and high-accuracy MS and MS/MS data [38,39,40]. Xevo G2 QTOF was applied for the identification of antioxidant non-volatile compounds (terpenoids, lactones, dihydrochalcones, ceramides, and diacylglycerols) from the fraction of industrial annatto (*Bixa orellana* L.) seed residue [41]. Tribrid systems (Orbitrap Fusion [42,43] or Eclipse [44,45]) offer enhanced analytical flexibility and are particularly effective for the structural characterisation of complex biomolecules [46]. In recent years, ion mobility (IMS), integrated with a mass spectrometer (e.g., TOF, quadrupole, Orbitrap, FT-ICR), has gained prominence in metabolomics due to its ability to address the challenges posed by complex food matrices, such as those derived from agri-food waste [47,48]. Despite challenges like sensitivity and matrix effects, ongoing improvements in instrumentation and data processing make IMS a valuable complement to chromatography and mass spectrometry in food metabolomics.

#### 3.1.2. MS Scanning Modes for Untargeted Analysis

Tandem mass spectrometry (MS/MS) is widely utilized in untargeted metabolomics due to its ability to provide structural information about metabolites in complex mixtures. Molecules are selected based on their mass-to-charge ratio (precursor or parent ions) and then fragmented using methods such as collision-induced dissociation (CID), higher-energy collisional dissociation (HCD), or alternative fragmentation techniques [49].

The resulting fragment ions (daughter ions) provide significant insights into the molecular structure, functional groups, and substructures of the parent compounds [50].

The Q-Orbitrap, tribrid Orbitrap, and Q-TOF hybrid systems offer advanced acquisition modes, including DDA (data-dependent acquisition) and DIA (data-independent acquisition), which are ideal for metabolic profiling. Data-dependent acquisition (DDA) and data-independent acquisition (DIA) are the two primary strategies for MS/MS data collection in liquid chromatography-tandem mass spectrometry (LC-MS) untargeted metabolomics. Both approaches are designed to capture precursors (MS^1^) with a full-scan, which provides an overview of the metabolome by recording the *m*/*z* and relative abundances of all detectable ions in a sample, followed by MS^2^ scans to fragment them.

In DDA, only the most intense ions are sequentially isolated for fragmentation, allowing for an easy correlation of each precursor to its corresponding MS/MS (MS^2^) spectrum, which facilitates structure elucidation [51]. Zalidis et al. (2025) applied DDA to analyse phenolic metabolites present in flours derived from agricultural food waste, focusing on grape seed and olive stone flours [50].

Conversely, DIA isolates and fragments all precursor ions within a defined *m*/*z* window, enabling MS^2^ data collection for all sample ions, which generates complex MS^2^ spectra; however, it lacks the direct precursor–fragment correlation observed in DDA [52]. These characteristics significantly complicate the spectral deconvolution and accurate identification of the metabolites. In the last decades, the SWATH-MS (sequential window acquisition of all theoretical mass spectra) has been developed, which partitions the mass range into sequential isolation windows (swaths) for the unbiased fragmentation of all detected ions. Since then, swath-based DIA has revolutionised metabolomics with high throughput, deep metabolite coverage, and reliable data [53,54]

#### 3.1.3. Targeted Metabolomics

While untargeted metabolomics aims to provide a comprehensive overview by detecting as many metabolites as possible within a sample, including unknown compounds, targeted metabolomics is designed to selectively quantify predetermined groups of metabolites with high specificity and sensitivity [55].

In quantitative metabolomics, it is common practice to measure the relative abundances of the analytes across samples in comparative studies. In contrast, absolute quantification requires standard calibration curves to precisely determine the concentration of specific metabolites within a sample, regardless of the experimental conditions [56].

Triple quadrupole (QQQ) [57] instruments have historically served as the benchmark for quantitative analysis due to their heightened sensitivity and the precise quantification facilitated by the multiple reaction monitoring (MRM) method (Table 1). This methodology provides robust analytical stability and efficiency in tracking specific precursor–product ion transitions (*m*/*z* of the precursor ion (Q1) and *m*/*z* of the product ion (Q3)).

Cuffaro et al. (2023) [58] quantified the polyphenols in olive mill wastewater, identifying oleacein and hydroxytyrosol as the most abundant metabolites, along with tyrosol, oleuropein derivatives, ligstroside aglycones, and verbascoside. These extracts showed strong antioxidant and anti-inflammatory properties, supporting their nutraceutical applications.

One crucial phase in the MRM process is the pre-optimisation of the MS settings, and fine-tuning parameters (such as collision energy and declustering potential [59]) requires the use of related chemical standards [60]. Consequently, MRM is essentially restricted to the analysis of known metabolites for which established transitions and standards are available [61].

By comparison, as Q-Orbitrap and Q-TOF technologies for high-resolution mass spectrometry, widely applied in untargeted metabolomics, have also shown promise for targeted metabolomics, thanks to the parallel reaction monitoring (PRM) scanning mode (Table 1) [62].

In this approach, the metabolite precursor ions are selected in the first quadrupole through an ion inclusion list and then fragmented using HCD or CID [63]. Simultaneously, all resulting fragment ions are detected in a single high-resolution scan. One significant advantage of PRM is the reduced dependence on chemical standards throughout the method development process. Even in the absence of a reference substance, high-resolution MS/MS spectra allow for the confident identification of metabolites based on mass accuracy and fragmentation patterns.

Targeted mass spectrometry is a powerful analytical tool for the precise quantification of bioactive metabolite classes, such as polyphenols [21], anthocyanins [64], or betains [65], in complex plant waste matrices. This facilitates the valorisation of agricultural byproducts and supports the identification of high-value compounds with potential applications in cosmetic formulations [66].

#### 3.1.4. Software for LC-MS Data Analysis in Plant By-Products

The great complexity and variability of LC-MS data obtained from the analysis of plant waste extracts require the use of sophisticated computational methods to ensure accurate data processing and reliable metabolite identification. To enable every stage of the analytical process, including peak detection, normalisation, structural annotation, statistical analysis, and functional interpretation, a wide spectrum of open-source and registered software solutions has been developed in recent years (Table 2). Using spectral databases, current open-source systems, including XCMS, Mzmine [67], MS-DIAL [68], Skyline [69] and OpenMS [70] provide substantial feature extraction and possible identification capabilities for untargeted metabolomics. Sometimes, these instruments are incorporated into more comprehensive processes using Galaxy, such as Workflow4Metabolomics, or through MetaboAnalyst’s integration within Galaxy [71]. By offering large MS/MS spectrum libraries and open-source databases, like METLIN [71] or FoodBD (www.foodb.ca) these tools facilitate the identification of compounds. Commercial applications include Progenesis QI (Waters) [72] and Compound Discoverer (Thermo Fisher Scientific) [73], as well as MetaboScape (Bruker) and MarkerLynx.

While open-source tools like XCMS, Mzmine, and MS-DIAL are preferred for their accessibility and flexibility, commercial platforms such as Progenesis QI (waters^TM^) and Compound Discoverer (Thermo Fisher Scientific^TM^) offer robust, user-friendly solutions with dedicated vendor support, although at a higher cost. MS-DIAL distinguishes itself by incorporating large MS/MS libraries, which are particularly valuable for metabolite identification. Databases such as METLIN and HMDB offer comprehensive coverage of compounds, including classes like phenolics, flavonoids, and terpenes, which are crucial for structural elucidation in plant-based samples. MassBank, GNPS, and FoodDB also play important roles in matching MS/MS spectra and contextualising results in food metabolomics, while LIPID MAPS and Lipid Blast are databases used for lipid identification [44].

## 4. Sample Preparation for LC-MS Analysis of Natural Products

Extraction is a crucial step in identifying and characterising bioactive compounds from natural sources. Sample preparation for liquid chromatography-mass spectrometry (LC–MS) typically involves isolating analytes from complex matrices, such as plant tissues. Various strategies can be employed to address challenges related to matrix complexity, low analyte concentrations, and sample heterogeneity [74].

One of the main challenges in LC-MS analysis of complex matrices is the susceptibility to matrix effects, which can suppress or enhance ion signals, ultimately affecting analyte ionization efficiency and overall method performance. Depending on matrix complexity, these effects can be minimised or eliminated by optimising sample extraction and cleanup procedures. Inadequate purification can lead to pronounced matrix effects; therefore, the cleanup process should aim to maximize analyte recovery while minimising matrix interferences [75].

The selection of an appropriate extraction solvent is critical for comprehensive LC-MS analysis, as no single solvent can dissolve all metabolites from plant matrices. Both the type of solvent and the solvent-to-sample ratio significantly influence extraction efficiency. The choice of solvent should align with the chemical properties of the target compounds and the analytical technique employed. Since metabolomic studies aim to detect the widest possible range of metabolites within a biological sample, solvents capable of extracting diverse metabolite classes are preferred. Additionally, the solvent must be compatible with the specific analytical platform, and generally, LC-MS allows for greater flexibility in solvent choice [76].

**Figure 3:**
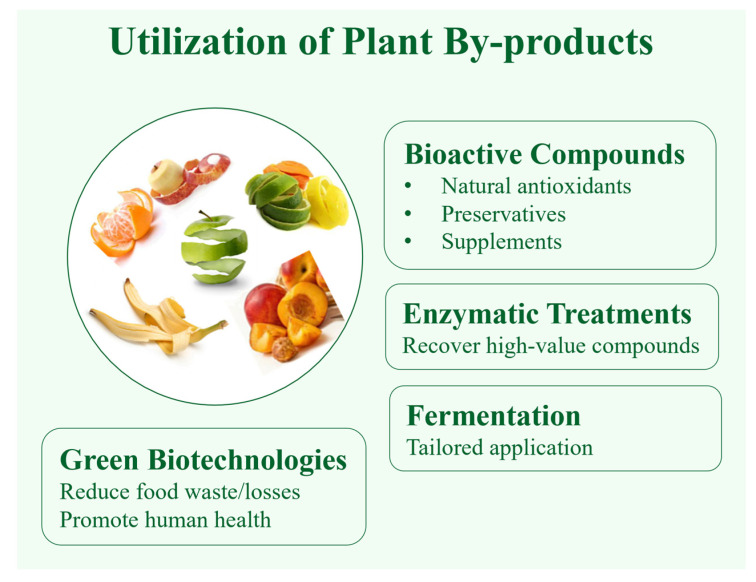
Use of bioactive compounds from vegetable and fruit waste.

Different extraction protocols are selected based on the physicochemical properties of the metabolites—whether polar or apolar—and the type of analytical approach employed. Untargeted analyses require broad, non-selective extraction methods to capture a wide range of metabolites, while targeted analyses benefit from more selective and optimized protocols tailored to specific compounds of interest (Figure 3) [77].

Single- and multi-step solid-phase extraction (SPE) has become a crucial technique for isolating and purifying analytes in various fields, including biomedical research, environmental monitoring, and food science. Moreover, conventional extraction methods are increasingly being replaced by greener, more sustainable alternatives [16].

**Figure 4:**
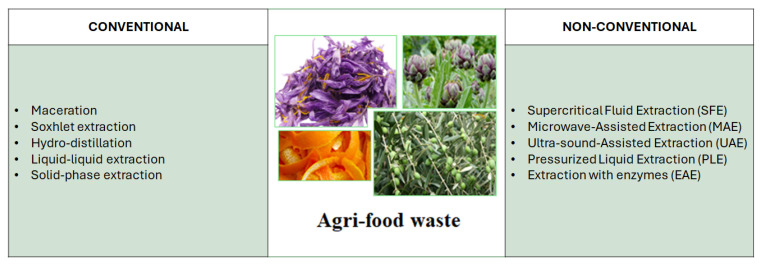
Conventional and unconventional extraction methods.

The most popular strategies for separating bioactive compounds from fruit and vegetable waste include conventional extraction methods such as maceration, Soxhlet extraction, hydrodistillation, liquid-liquid extraction, and solid-phase extraction (Figure 4).

However, these methods present several challenges, including low efficiency, high costs, degradation of natural compounds, and potential health risks [13].

Some characteristics, such as viscosity, purity, selectivity, density, toxicity, volatility, reactivity, and miscibility with aqueous media, affect the efficacy of conventional extraction techniques that utilise organic solvents, including maceration, hydrodistillation, and steam distillation. For this reason, they have been replaced by unconventional methods [14].

The goal of unconventional methods, also known as green extraction, is to develop innovative methods that use sustainable natural resources, safe and non-toxic alternative solvents, and require less energy. Green extraction processes are reported to offer higher yields, lower solvent consumption, and greater efficiency [15,78]. In these environmentally friendly extraction approaches, the selection of the solvent is crucial. Solvents should be entirely natural, non-toxic, biodegradable, and ready for implementation [79].

Natural deep eutectic solvents (NADES) are a green solution to conventional organic solvents, offering high extraction efficiency for plant phytochemicals. NADES consist of a hydrogen receptor and natural hydrogen donors, overcoming toxicity and environmental concerns [24]. NADES (natural acid dehydrogenase) and MAE (mixed alkyl ether) have been employed for the extraction of polyphenolic chemicals from diverse plant waste matrices, such as olive oil, winemaking, brewing, fruit and vegetable processing, and medicinal plants. NADES are an effective alternative to traditional solvents for the recovery of phenolic components from waste matrices, yielding better extraction results [80,81]. Another type of green solvent that can be utilised with green technology to recover natural products and prevent the production of hazardous effluents is DESs [25].

Unconventional methods include supercritical fluid extraction (SFE), microwave-assisted extraction (MAE); ultrasound-assisted extraction (UAE), extraction with pressurized liquids (PLE—pressurized liquid extraction) and extraction with enzymes (EAE) (Figure 4).

Of course, these unconventional extraction methods have both advantages and disadvantages.

- Microwave-assisted extraction (MAE) offers speed, efficiency, and low cost. However, it is not suitable for thermally labile compounds.
- Enzyme-assisted extraction (EAE) is highly efficient, selective, eco-friendly, and can be performed at low temperatures. Nonetheless, it requires expensive enzymes and is a time-consuming process.
- Ultrasound-assisted extraction (UAE) is user-friendly, efficient, environmentally friendly, and allows versatile solvent selection. Its drawbacks include the need for multiple extractions and the potential generation of radicals.
- Supercritical fluid extraction (SFE) is non-toxic, cost-effective, and rapid. Additionally, the supercritical fluid can be recycled. However, it has a high initial cost and is mainly limited to non-polar phytochemicals.
- Pulsed electric field (PEF) requires shorter extraction times and increases cell permeability, but the equipment is costly to maintain.
- Pressurized liquid extraction (PLE) is energy-efficient, uses non-toxic solvents, and requires simple equipment. However, it has high equipment costs and may lead to the inactivation of certain compounds [82].

## 5. Bioactive Compounds Detected by LC-MS in Plant Waste

Recent research suggests a potential link between food waste and the cosmetics industry. Compounds derived from food waste are being used by numerous natural cosmetic companies [83,84,85,86]. Food waste, comprising fruit peels, seeds, and vegetable waste, contains phenolic compounds, flavonoids, and organic acids that possess antibacterial, antioxidant, and anti-inflammatory characteristics [87,88,89,90]. Carotenoids, enzymes, polyphenols, lipids, vitamins, and other biomolecules with a variety of health-promoting properties are among the many beneficial bioactives found in by-products of fruit processing [21].

Liquid chromatography coupled with mass spectrometry is one of the primary analytical techniques used for identifying polyphenols in plant waste, as confirmed by several studies (Table 3) [91,92,93,94,95]. Polyphenols are categorised into different classes based on the number of phenolic rings and the substitution patterns.

A study focused on wine lees, using green processing, yielded a concentrated solution rich in phenolic components, especially hydroxycinnamic acids. Caftaric and coutaric acids have been reported as the predominant components, in addition to hydroxybenzoic acids, gallic acid, and 2,5-dihydroxybenzoic acid. The flavonoids identified were astilbin and catechin [91].

Further studies employing LC-MS have demonstrated that olive leaves and olive mill wastewater are an abundant source of bioactive polyphenols with nutraceutical properties [58,92]. A study by Houasni et al. (2022) [92] optimised a simultaneous organosolvent treatment and extraction process at elevated temperatures (>110 °C), using glycerol and two glycerol-based deep eutectic solvents (DES). Liquid chromatography-mass spectrometry (LC-MS) analysis revealed that extraction with either DES resulted in a distinct polyphenol profile compared to extractions using water or 60% (*v*/*v*) aqueous ethanol. Notably, when an alkaline DES composed of glycerol and sodium citrate was used, hydrolysis of flavone glucosides was observed. This investigation suggested that high-temperature organosolvent extraction, which requires shorter extraction times than conventional methods, could be an effective and environmentally sustainable approach to valorising olive leaves and developing value-added products.

Liquid chromatography-mass spectrometry has also been utilised in recent efforts to characterize the polyphenolic composition of saffron tepals, particularly to evaluate green extraction processes that combine two subcritical water extraction steps with an enzymatic treatment incorporated between them [96].

Anthocyanins are polyphenolic compounds that give fruits and vegetables their colour and attractive appeal; they can also be separated and detected by LC-MS. They can be extracted from various vegetables and fruits, such as red onions, berries, and pomegranates, and are among the main compounds that can be recovered from food waste [97,98]. A noteworthy recent study involved the application of acid whey as a bio-based solvent for extracting anthocyanins from red cabbage waste. Acid whey is a promising bio-based solvent that can enhance the value of agro-industrial by-products [99]. This work aimed to obtain an anthocyanin-rich extract from red cabbage by-products using acid whey as the solvent and employing advanced extraction techniques, specifically ultrasound-assisted extraction (UAE) and pressurized liquid extraction (PLE).

The extraction experiments were conducted to assess the quantity and quality of extracts obtained from each method. Two solvents were employed for each extraction technique: acid whey (AW) and acidified ethanol (EA).

The phenolic profile of red cabbage leaf extracts was determined using LC-DAD-ESI/MS^n^. Individual anthocyanins were identified using a DAD and a linear ion trap LTQ XL mass spectrometer. From this analysis, it was observed that both UAE and PLE provided higher yields compared to the conventional method [99].

LC-MS analysis was also employed for the detection and quantification of carotenoids in various fruit waste, such as papaya and tamarillo [100,101], and to identify and quantify carotenoids in discoloured red peppers [102].

The extraction of carotenoids from plant waste is challenging due to their low concentration, strong binding within complex plant matrices, and sensitivity to heat, light, and oxygen. Several studies have investigated advancements in carotenoid extraction techniques, focusing on environmentally sustainable recovery approaches for high-value applications, such as functional foods and pharmaceuticals [101]. Nagarajan et al. (2020) [103] investigated carotenoid–pectin complexation as a promising green approach for extracting valuable carotenoids from tomato pomace. The carotenoid and pectin recovered from the complexation process were structurally analysed by high-performance liquid chromatography (HPLC) and spectroscopy.

Vitamins, mainly tocopherol, ascorbic acid, and vitamin K, are other potential sources that have been reported to be extracted from plant waste and analysed by LC-MS [100,104]. Based on their solubility, vitamins are categorised as either fat-soluble (A, D, E, and K) or water-soluble (B and C).

Porter et al. (2021) investigated the extraction efficiency of vitamer K1 from plant materials using pressurised liquid extraction (PLE) and ultrasound-assisted solvent extraction (UASE) with LC-MS analysis, highlighting the importance of efficient, economical, and ecological methods for extracting and analysing vitamin K in complex matrices [105].

Carotenoids are other bioactive compounds abundant in food waste, especially tomato waste [106], and offer various health benefits. Recently, a study has developed an efficient recovery protocol for carotenoids (β-carotene and α-carotene) from carrot waste using eco-friendly solvents. The effectiveness of 2-methyltetrahydrofuran (2-MeTHF), limonene (Lim), and cyclopentyl methyl ether (CPME) in extracting carotenoids was compared with ethyl acetate (EtOAc), a conventional green solvent commonly used to replace hexane and petroleum ether [107].

The authors demonstrated positive biological activities and the biocompatibility of these recovered carotenoids for use on human skin as an anti-aging agent, indicating their potential application in the cosmetic industry [107].

**Table 3:** Examples of plant waste extracted with conventional and non-conventional methods and analysed by liquid chromatography-mass spectrometry with the identification of different classes of compounds.

Plant Waste	Extraction Methods	Analytical Techniques	Bioactive Compounds	Ref.
Wine lees	Maceration followed by microfiltration step	UHPLC-LTQ Orbitrap and Velos LC-QTrap 4000	Phenolic compounds	[91]
Olive leaves	Organo-solv extraction	LC-Finnigan AQA	Polyphenols	[92]
Tomato pomace	Maceration	LC-Q-Exactive	Polyphenols	[93]
Spent black tea	Pressurised liquid extraction	LC-LTQ-Orbitrap XL	Phenolic compounds	[94]
Aloe vera leaf	UAE and MAE extractions	LC/MS single quadrupole	Anthraquinone and chromone derivatives	[95]
Olive mill wastewater	Liquid-liquid extraction	LC-QTRAP 6500	Polyphenols	[58]
Saffron tepals	Enzymatic and subcritical water extractions	LC-LTQ XL linear ion trap	Flavonoids	[96]
Red cabbage waste	Pressurized liquid extraction (PLE), ultrasound-assisted extraction (UAE), and heating and stirring extraction (HSE)	LC-Linear Ion Trap	Anthocyanyns	[99]
Annatto by-product	Maceration	UPLC-MS/QTOF Analysis	Terpenoids, ceramides	[41]
flower *P. serrulata*	Maceration	LC-Q-Trap 6500 in MRM mode	Anthocyanins	[64]
Plum seed residue	Matrix solid-phase dispersion	HPLC-ESI-QTOF-MS	Phenolic compounds	[37]
Walnut husk and pellicle	Ultrasound	UHPLC-Q-Exactive	Flavonoids, tannins and quinones	[38]
Chia leaves	Maceration	UHPLC-Q-Exactive	Polyphenols	[39]
Peach by-product	Extraction with enzyme (EAE)	UPLC-Qexactive	Phenolic acid Flavonoids	[40]
Mustard, kale, and broccoli microgreens	Maceration	UHPL-Fusion Orbitrap	Flavonoids	[43]
Foxnut	Maceration	LC-Orbitrap eclipse	Lipids, amino acids, phenolic acid Flavonoids alkaloids	[44]
Roots of red beet and sugar beet	Maceration	LC-LTQ XL linear ion trap	Betalins Phenolic compounds	[59]
Peel of *Citrus retigulata*	Ultrasound extraction	LC-Q-Exactive	Flavonoids	[63]
Fennel waste	Ultrasound extraction	UPLC-Qtrap 6500	Flavonoids	[66]
Apple cider by-products	Maceration	LC-Q-TOF	Carotenoids, phenols	[85]
Tamarillos	Maceration	LC-APCI-MS/MS	Carotenoids	[100]
Red pepper skin	Maceration	LC-Q-Exactive	Carotenoids	[102]
Avocado	Maceration	UHPLC-Q-Tof	Phenolic acids	[108]
Mango peel	Maceration	LC-ESI-MS	Polyphenols	[109]

## 6. Bioactive Compounds from Plant Waste and Their Potential Application in Cosmetics

The physiological and morphological characteristics of the skin are constantly evolving, influenced by various internal and external factors that contribute to skin issues, such as inflammation, aging, cancer, oxidative stress, and hyperpigmentation. Plant-based skincare products are receiving increased attention due to their safety compared to synthetically generated cosmetics. Numerous in vitro and in vivo investigations have elucidated the therapeutic efficacy of plant extracts with dermatological relevance [110] (Table 4).

The concentration of polyphenols is often higher in the waste produced from fruit and vegetables than in the edible parts, since phenolic acids, flavonoids, and tannins remain present [111]. Thanks to their redox potential, flavonoids and anthocyanins act as reducing agents and hydrogen quenchers, which makes them very antioxidant [112]. Many flavonoids, such as catechol and gallol, found in tea can act as chelating agents for metal ions, thereby offering protection against heavy metal toxicity [113]. Polyphenols found in plants recovered from agro-industrial waste offer numerous health benefits such as anti-carcinogenic, anti-hyperpigmentation, antioxidant, anti-aging, antibacterial, and anti-inflammatory effects [114]. Flavonoids, such as quercetin, kaempferol, and myricetin, effectively block histamine release and may be considered cosmeceutical agents due to their capacity to enhance skin–blood microcirculation [113].

Polyphenols serve as a promising photoprotective agent in the prevention of skin damage and melanoma induced by damaging radiation. They possess UV-absorbing characteristics, efficiently obstructing UV light from penetrating the stratum corneum when applied topically [115].

These components also act as natural sunlight protectors due to their antioxidants, anti-inflammatory and ROS scavenging properties, offering significant photoprotection effects. Polyphenols have antioxidant properties, inhibiting lipid peroxidation, reducing UV-induced nitric oxide and hydrogen peroxide levels, and regulating the cellular redox state. They have been shown to enhance cell survival rates, induce apoptosis, and prevent tumours [116]. There are numerous benefits associated with polyphenols, including skin photoprotection, suppression of photocarcinogenesis, and antioxidant capabilities. These discoveries may be applicable in several medical fields, including cosmetics and skincare items [110].

Among the polyphenols, anthocyanins are a class of compounds that have been reported to prevent skin hyperpigmentation by absorbing UV light and inhibiting tyrosinase, a crucial enzyme involved in melanin synthesis, and they could be a promising candidate as skin-whitening agents in cosmetic formulations [117].

Lutein, which is found in dark green leafy vegetables, is one of the most common xanthophylls, while beta-carotene is found in orange fruits and vegetables [111]. Due to their chemical structure, characterised by a series of conjugated double bonds, carotenoids act as antioxidants, and may play a role in preventing chronic diseases, including various types of cancer [118,119,120]. Despite their antinutritional effects, tannins also play an important antioxidant role due to the multiple phenolic hydroxyl groups in their chemical structure. They can also exert antimicrobial activity by inhibiting enzymatic activities, depleting metal ions, and precipitating membrane proteins of microorganisms [121].

Ascorbic acid and tocopherol, along with their derivatives, are the most prevalent antioxidants in anti-aging formulations [122]. Ascorbic acid is an essential nutrient with significant promise as a cosmeceutical for anti-aging skin applications. It is reported to mitigate photoaging and intrinsic aging of the skin by diminishing oxidative stress from both external and internal sources, and by enhancing collagen gene expression and maturation [123].

Among the vitamins mentioned above, vitamin K has been studied as a wound healing treatment; however, it is challenging to draw definitive conclusions regarding its potential use in this context [124].

Phytosterols, which belong to the triterpene family, are found exclusively in plants. They are present in various food by-products, such as wheat bran, rice bran, wheat germ, and oat bran, all of which are by-products of grain processing. Non-polar extracts of these by-products can also be a rich source of phytosterols [125]. Phytic acid, found in several plant by-products, including cereals, legumes, vegetables, and nuts, has recently gained attention for its anti-inflammatory activity [126]. Phytic acid is a natural product, widely used as a depigmenting agent in cosmetic emulsions [127]. Waste products from the agri-food industry contain significant amounts of dietary fibre, which can be classified into two groups based on its physical, chemical, and functional properties: water-soluble fibre (e.g., pectin) and water-insoluble fibre (e.g., cellulose and lignin) [128]. Pectin is a natural polysaccharide and is a versatile ingredient in cosmetics. It acts as an emulsifier, viscosity regulator, and stabiliser. Sources of pectin include citrus fruits, such as lemons and pomelos, as well as beetroot and banana peels [129].

Lipids are chemically diverse compounds that are insoluble in water yet soluble in non-polar solvents. They participate in numerous essential cellular functions, encompassing biological membranes, energy storage, and signalling mechanisms. Lipids and their derivatives serve various functions in cosmetic formulations, including moisturising, emollient, and softening agents; surfactants and emulsifiers; providing product consistency; acting as carriers for colour and fragrance; preserving product integrity; and contributing to the molecular delivery system. Recently, there has been an increasing interest in utilising microalgal lipids in various sectors, including the cosmetic industry [130].

Bioactive protein hydrolysates and peptides recovered from food wastes have significant functional, nutraceutical, and cosmeceutical value [131].

In recent years, numerous studies have described bioactive peptides (biopeptides) as compounds of interest for industrial applications due to their numerous functional properties. (e.g., antioxidant, anti-aging, anti-inflammatory, and antimicrobial properties) and technological properties (e.g., solubility, foaming, and emulsifying properties). Peptides have gained worldwide attention for their sustainability, with fewer side effects than synthetic drugs [132]. According to the US Food and Drug Administration, peptides are considered amino acid polymers with a specific sequence and contain less than 40 amino acids in total. Waste-derived bioactive peptides (BPs) have health benefits, making them promising ingredients in the food, cosmetic, and pharmaceutical industries. BP have been widely used in the production of biopolymer-derived films for antimicrobial active packaging [133] to stabilize food/pharmaceutical matrices [134], and for the prevention of chronic diseases [135,136], and have very promising effects mainly on hypertension, diabetes mellitus (DM), hypercholesterolemia, inflammatory processes, immunity, and cancer [136]. Today, the cosmetic industry considers food peptides as innovative bioactive compounds for use in cosmetics production.

Based on their expected mechanism of action, cosmetic peptides can be classified as follows: signalling peptides, which stimulate the production of matrix proteins and cell growth, carrier peptides (which help transport active compounds into the cell); neuro-transmitter-inhibiting peptides (which inhibit the release of acetylcholine, which may lead to the appearance of expression lines); and enzyme-inhibiting peptides (which reduce the activity of enzymes related to skin aging) [137].

The growing demand for natural cosmetics has led to the development of a new generation of products based on biopeptides that can improve skin health by acting against enzymes linked to aging and reducing the harmful effects of agents that cause skin damage (acting as antioxidants, antimicrobials, and anti-inflammatories).

Pentapeptide-18 (Leuphasyl^®^), palmitoyl pentapeptide-4, acetyl hexapeptide-8 (Argireline^®^), and other synthetic peptides are currently used in cosmetics for their anti-aging properties. They mimic a naturally occurring protein fragment in the skin, stimulating the production of collagen and elastin and improving skin firmness by reducing the appearance of wrinkles [138]. Some natural peptides, such as those found in the skin of frogs, snake venom, yeast, spirulina, toads, and fish, have properties that can help reduce the visible signs of aging. Kafirins, which are obtained from the protein fraction of waste grain from white sorghum (*Sorghum bicolor* (L.) Moench), represent a suitable raw material for the enzymatic production of peptide extracts that protect the skin from UVB-induced damage (photoaging) [139]. Soya peptides are bioactive substances derived from soy proteins. Some of these peptides have strong antioxidant properties, which improve skin elasticity and hydration [140]. They help reduce damage caused by free radicals, contributing to a more youthful appearance by improving skin resilience and strength.

Several steps are required for the production of biopeptides from food waste, including purification (pretreatment, chromatography, physical methods, microbial fermentation, and enzymatic processes) and characterisation (NMR spectroscopy, mass spectrometry) [141]. Peptides and proteins are analysed using high-performance liquid chromatography (HPLC) and tandem mass spectrometry (MS) methodologies. Reverse-phase high-performance liquid chromatography (RP-HPLC) is suitable for analysing small peptides in plant matrices. In contrast, size exclusion chromatography (SEC) and ion exchange chromatography (IEC) may be employed in conjunction. Hydrophilic interaction liquid chromatography (HILIC) is increasingly used to isolate highly polar molecules, thereby increasing the quantity of discovered peptides. ESI-MS detects femtomole quantities of compounds, including peptides. It can analyse the intact mass and amino acid sequence of peptides. Generally, MALDI-TOF/TOF instrumentation can be applied to obtain more specific and reliable results, providing evidence of their structures [136,141].

Cosmetic products are easily susceptible to bacterial and fungal contamination during their use, with the most common microbial agents including *Staphylococcus aureus*, *Escherichia coli*, and *Pseudomonas aeruginosa* among bacteria, and *Candida albicans* among fungi. Plant-based waste materials are often used to obtain extracts with antimicrobial properties, and due to their antibacterial activity, these plant-derived extracts may serve as natural preservatives in cosmetic formulations [86,142].

Different types of food waste have been shown to exhibit antimicrobial activity. For example, olive leaf extracts have demonstrated activity against various bacteria, including *Listeria monocytogenes*, *Escherichia coli*, *Staphylococcus aureus*, and *Pseudomonas aeruginosa* [143]. Similarly, orange peel extracts showed antimicrobial effects against *Staphylococcus aureus*, *Enterococcus faecalis*, *Pseudomonas aeruginosa*, and *Escherichia coli* [144].

In a recent study, Prevete et al. (2024) [145] prepared aqueous extracts of olive leaves and orange peels, both rich in phenolic compounds, using ultrasound-assisted extraction. Each extract was then incorporated into liposomes. The antimicrobial capacity of both the free extracts and the liposome-encapsulated forms was evaluated against various strains of potential bacterial pathogens. The results demonstrated that encapsulation of the olive leaf extract in liposomes significantly enhanced its antibacterial activity against *Staphylococcus aureus*.

Among the phytochemicals extracted from plant sources, catechins are a category of polyphenolic compounds found in several plant sources, including green tea and fruits such as grapes, apples, pears, and cherries [146]. They exhibit various biological activities, including antibacterial, antioxidant, anti-inflammatory, and therapeutic properties, and have demonstrated efficacy in the healing of burns, the treatment of diarrhoea, and the alleviation of cancer sores. Due to their antihyperlipidemic, thermogenic, anticarcinogenic, and probiotic properties, catechins are a valuable raw ingredient in cosmetics. Recent research highlights the potential of these compounds as cosmetic ingredients due to their antibacterial properties [146]. A variety of formulations using catechins have been created, including catechin-infused soaps and transparent soap formulations. These advancements have created numerous opportunities for cosmetic formulation, including anti-acne creams and catechin-based sunscreens [146,147].

Catechins act against bacteria in several ways: they can disrupt cell walls and membranes through polyphenol reactions, and also adhere to cell membranes, blocking essential mechanisms for bacterial proliferation, such as altering peptidoglycan biosynthesis or inhibiting the formation of penicillin-binding protein 2. The production of biofilms by various types of bacteria, including *S. mutans* and *E. coli*, can be inhibited by catechins, which also hinder bacterial development [148].

Investigations into the antibacterial properties of catechins have demonstrated that natural deep eutectic solvents (NADES) can enhance the effectiveness of antimicrobial agents against four distinct types of catechins: epicatechin (EC), epicatechin, epigallocatechin (EGC), and epigallocatechin-3-gallate (EGCG). Choline chloride/glycerol (ChG) was shown to enhance the thermal stability and storage of catechins [149].

For this reason, catechins are a promising ingredient for cosmetic purposes, as they have antibacterial properties and can inhibit bacteria such as *Staphylococcus aureus*, *Pseudomonas aeruginosa*, *Enterobacter aerogenes*, and *Candida albicans*. They are also known for their UV protection, anti-inflammatory, and antioxidant properties [146].

Bioactive peptides represent a class of emerging compounds derived from food waste, with potential applications as natural preservatives in cosmetic formulations. Notably, peptides isolated from millet grains demonstrated antibacterial activity and showed no cytotoxicity toward endothelial cells [150].

**Table 4:** An overview of interesting bioactive compounds detected in plant waste and their possible cosmetic applications.

Plant Matrix	Bioactive Compounds	Activity	Cosmetic Applications
*Rosmarinus officinalis* (rosemary) leaf extracts [151]	Phenolic, flavonoidic compounds	Antimicrobial	Natural preservatives
*Coffea arabica* and *Coffea canephora* by-products [152]	Terspens, alkaloids, phenolic acids	Antimicrobial	Natural preservatives
*Prunus* leaves [153]	Tannins	Antioxidant and lipid peroxidation inhibitory activities	Anti-aging
*Horse chestnut* flower [154]	Flavanols derivatives, phenolic acids, flavanols	Antioxidant, ROS scavenging.	Anti-aging, antioxidant serums
*Camellia sinensis* flowers [155,156]	Flavonols, Catechins, Caffeine, Theanine, triterpene saponins	Antioxidant, Anti-inflammatory, Anti-obesity,	Anti-aging creams Skin-soothing products Slimming and UV-protection boosters, hair protection
*Eucalyptus globulus* leaves [157]	Polyphenols (Gallic acid, 5-caffeoylquinic acid, ellagic acid, ellagitannins, quercetin derivatives, and luteolin 7-*O*-glucuronide)	Anti-aging	Polyphenols (gallic acid, 5-caffeoylquinic acid, ellagic acid, ellagitannins, quercetin derivatives, and luteolin 7-*O*-glucuronide)
*Harpagophytum procumbens* (devil’s claw) [158]	Verbascoside, leucosceptoside A	Anti-inflammatory	Treatment for psoriasis-prone skin, soothing or skin-repairing formulations
*Lycium barbarum* (seed dreg) [159]	Polysaccharides	Antioxidant protection, hydration support	Polysaccharides
*Mentha × piperita* L. [160]	Flavonoids, phenolic acids, triterpenoids, hydroxybenzoic acids, hydroxycinnamic acids	Antioxidant, anti-inflammatory, antimicrobial, immunomodulatory, hepatoprotective, antiviral	Anti-aging, relieving, oxidative stress protection,
Ginseng root [161]	Ginsenosides, lignans, glycosides, polyphenols	Antioxidant, anti-inflammatory, collagen synthesis promotion, skin barrier support	Anti-aging, skin hydration, skin barrier protection
Olive fruit, leaves, and byproducts [114]	Phenolic acids, phenolic alcohols, flavonoids	Antioxidant, anti-inflammatory, antimicrobial, antiproliferative	Anti-aging, antioxidant skin care, anti-inflammatory formulations, skin barrier protection
Tomato processing waste (pomace: peel and seeds) [106]	Carotenoids (lycopene), phenolic compounds, vitamins, flavonoids	Antioxidant, antimicrobial, anti-inflammatory, antithrombotic, glycaemic regulation, cardiovascular protection	Antioxidant skin protection, anti-aging, preservatives
Olive leaf meal and spent *Pleurotus ostreatus* substrate [118,162]	Carotenoids	Antioxidant, provitamin A activity	Photo-protective
Pomegranate (*Punica granatum* L.) peel and peel extract [121]	Polyphenols, flavonoids, tannins, ellagitannins, anthocyanins	Antioxidant, anti-inflammatory, antimicrobial, radical scavenging, UV-protective	Anti-aging creams, UV protection, anti-wrinkle, soothing lotions, and acne treatment
Blackcurrant pomace [163]	Pectin polysaccharides	Antioxidant activity Antimicrobic	Prebiotic cosmetic ingredients, emulsifiers
Tomato, apple, guava, dates seeds [134]	Proteins (bioactive peptides), carotenoids, polysaccharides (pectin), flavonoids, vitamin.	Antioxidant, antibacterial, anti-inflammatory	Skin aging and skin hydration and elasticity
*Camellia sinensis*, *Uncaria gambir* Roxb, *Canarium patentinervium* Miq, Grapes, apples, pears, cherries [147]	Catechins, flavonoids	Antioxidant, antimicrobial anti-inflammatory	UV protection, anti-aging, wound healing, sunscreen

## 7. Materials and Methods

References pertinent to the subject of the present review study were sourced from the Scopus, Google Scholar, and SciFinder databases. The years 2020–2025 were mainly used as the reference period, and only papers in English were included. The search strategy encompassed the following phrases and words: “agri-food waste”, “LC-MS”, “NMR”, “metabolite profiling”, “cosmetics” OR “cosmetic products”, “food waste”, “bioactive compounds”, “plant waste”.

## 8. Conclusions

In conclusion, current developments in LC-MS-based metabolomics have significantly improved the investigation of fruit and vegetable by-products, enabling the identification and quantification of bioactive metabolites with potential applications in cosmetics. This analytical technique enables the comprehensive characterisation of complex metabolite mixtures, thanks to its remarkable sensitivity and specificity, which allow for the identification of bioactive compounds, such as polyphenols, flavonoids, and terpenoids, that may contribute to desirable antioxidant, anti-inflammatory, or skin-rejuvenating effects in cosmetic formulations.

Upcycling plant-based by-products in cosmetic and cosmeceutical products aligns with sustainability and the circular economy. If reused, food waste rich in bioactive compounds offers multiple advantages:

- Reduction of food waste: food processing residues can be transformed into functional nutraceuticals or cosmeceuticals, thereby minimising environmental impact.
- Convenient nutraceutical integration: plant by-products are an economical source of compounds with diverse biological properties.
- Ecological and renewable resources for cosmetics: unlike synthetic antioxidants and antimicrobials, bioactive compounds of plant origin are naturally occurring and biodegradable.

Leveraging LC-MS-based metabolomics to analyse plant-based waste provides a powerful and precise method for identifying valuable bioactive compounds. This advanced technique not only facilitates the efficient recovery of natural ingredients but also drives sustainable innovation in the cosmetics industry, in line with the growing consumer demand for eco-friendly and clean-label products.

## Figures and Tables

**Table 1:** Mass spectrometers predominantly utilised for metabolomics analysis, the corresponding type of mass analysers employed, and the potential acquisition modes. Acquisition modes indicated by an asterisk * are primarily employed in quantitative workflows and are thus more relevant to targeted metabolomics.

Instrument	Configuration	Acquisition Modes
Thermo Scientific Q Exactive (Q Exactive, Q Exactive Plus, Q Exactive HF-X)	Quadrupole + Orbitrap	Full MS, DDA, DIA, PRM *
Thermo Scientific Orbitrap Exploris (Exploris 120, 240, 480)	Quadrupole + Orbitrap	Full MS, DDA, DIA, PRM *
Thermo Scientific Orbitrap Tribrid (Fusion, Fusion Lumos, Eclipse, ID-X, IQ-X)	Quadrupole + Ion Trap + Orbitrap	DDA, MS^n^
Agilent Q-TOF (6545, 6546)	Quadrupole + Time-of-Flight	DDA, DIA, SWATH, MS/MS
Waters Xevo QTOF (G2-XS, G3-XS)	Quadrupole + Time-of-Flight	DDA, DIA
Bruker timsTOF (timsTOF Pro, timsTOF Flex)	Quadrupole + TIMS + Time-of-Flight	DDA, DIA
SCIEX ZenoTOF 7600	Quadrupole + Zeno Trap + Time-of-Flight	DDA, SWATH, MRM^HR *

DDA: Data-dependent acquisition; DIA: Data-independent acquisition; MRM: Multiple reaction monitoring; PRM: Parallel reaction monitoring; MS: Mass spectra; SWATH: Sequential window acquisition of all theoretical fragment ions. Info companies: Thermo Fisher Scientific, Bremen, Germany; Waters Corporation, Milford, MA, USA; Agilent Technologies, Santa Clara, CA, USA; Bruker Corporation, Billerica, MA, USA; SCIEX, Concord, ON, Canada.

**Table 2:** Main software platforms currently used in metabolomics for plant waste analysis.

Software	Type	Functions	Supported Database	Notes
XCMS	Open-source	Peak detection, retention time alignment, feature extraction	METLIN, HMDB	Often used with METLIN for identification
Mzmine	Open-source	Peak picking, deconvolution, alignment, quantification	MassBank, GNPS	User-friendly interface; commonly used in academic research
MS-DIAL	Open-source	Deconvolution, alignment, MS/MS-based identification	Built-in libraries, MassBank	Includes large MS/MS libraries
Skyline	Open-source	Targeted and untargeted analysis, quantification	HMDB, METLIN	Originally for proteomics, now also used in metabolomics
OpenMS	Open-source	Full pipeline support: preprocessing to statistical analysis	Various	Highly modular; used in bioinformatics workflows
Workflow4Metabolomics	Open-source	Full LC-MS data analysis pipeline (XCMS-based)	METLIN, HMDB	Integrated into Galaxy platform
MetaboAnalyst (Galaxy)	Open-source	Statistical, pathway, and functional analysis	Various	Integrated into Galaxy; requires preprocessed data
Progenesis QI	Commercial	Feature extraction, identification, statistical analysis	Vendor specific	Developed by Waters
Compound Discoverer	Commercial	Peak picking, identification, isotope/adduct analysis	Vendor specific	Developed by Thermo Fisher Scientific
MetaboScape	Commercial	Data processing and annotation supports ion mobility	Vendor specific	Developed by Bruker
MarkerLynx	Commercial	Data pre-processing and statistical analysis	Vendor specific	From Waters; integrated with their instruments

## Data Availability

Data are contained within the article.
